# Role of cystatin C in renal damage and the optimum cut-off point of renal damage among patients with type 2 diabetes mellitus

**DOI:** 10.3892/etm.2014.1815

**Published:** 2014-06-30

**Authors:** XIAOPANG RAO, MEIYAN WAN, CAIXIA QIU, CHONGCAI JIANG

**Affiliations:** 1 Department of Endocrinology, Chengyang People’s Hospital, Qingdao, Shandong 266109, P.R. China; 2 Department of Nephrology, Qingdao Municipal Hospital, Qingdao, Shandong 266100, P.R. China

**Keywords:** serum cystatin C, urinary cystatin C, type 2 diabetes mellitus, renal impairment, cut-off point

## Abstract

The aims of the present study were to evaluate the roles of serum cystatin C (SCysC) and urinary cystatin C (UCysC) in renal function impairment and investigate the optimum cut-off point for renal function impairment among patients with type 2 diabetes mellitus (DM). A total of 742 inpatients and outpatients with type 2 DM (age, 20–75 years) were enrolled in this population-based cross-sectional study. The levels of SCysC and UCysC were determined and the odds ratios (ORs) and 95% confidence interval (CIs) of the calculated risk ratios of the different renal damage indicators were obtained. The levels of UCysC, urinary β2-microglobulin (Uβ2-MG), urinary albumin (UALB) and SCysC in the renal function impairment groups were observed in the following order: GFR-C>GFR-B>GFR-A (P<0.05 or P<0.01). According to the levels of GFR were divided into 4 groups, group GFR-A ≥ 80ml/min, GFR-B group 50–80 ml/min, group Ccr-C 20–50 ml/min, group GFR-D <20 ml/min. Following adjustment for age and gender, multivariate correlation analysis results revealed that levels of Uβ2-MG, UCysC and UALB negatively correlated with the glomerular filtration rate (GFR; P<0.05 or P<0.01). In addition, the duration of DM and the levels of SCysC and serum uric acid were shown to positively correlate with the GFR (P<0.05 or P<0.01). ORs for early renal function impairment significantly increased from the DM duration category of four years (OR, 1.74; 95% CI, 1.54–1.92). Receiver operating characteristic analysis demonstrated that the optimum DM cut-off point was four years, in which 60.79% sensitivity and 69.66% specificity were observed. Therefore, UCsyC levels may be used as an efficient indicator for the evaluation of early renal function impairment among patients with type 2 DM. In addition, renal lesions may initially occur in the renal tubule and then form in the renal glomerulus of patients with type 2 DM.

## Introduction

Diabetic nephropathy (DN), a major chronic complication of diabetes mellitus (DM), is considered to be one of the leading causes of end-stage renal failure (1). Previous studies on type 1 DN have shown that DN usually leads to microvascular diseases, including glomerular and tubular glomerular hypertrophy, thick glomerular membrane extracellular matrix accumulation and tubule-interstitial sclerosis (2,3).

DN is relatively obscure and progressive, thus, the disease is difficult to detect as traditional detection methods are less sensitive, particularly at early stages. Once patients suffer from proteinuria, DN is predominantly at an irreversible stage, posing a serious threat to the patients’ life (4,5). Therefore, early diagnosis of DN is extremely important. The commonly used clinical indicators for DN diagnosis are creatinine (CCr) and urea nitrogen. Various factors, including gender, height, muscle mass and diet, are associated with DN. Studies have also assessed renal damage and various indicators, including serum CCr (SCr), blood urea nitrogen, serum uric acid (SUA), β2-microglobulin (β2-MG), urinary albumin (UALB) and hematuria cystatin C (CysC). However, inconsistent results have been produced with regard to the sensitivity of type 2 DN, and the onset time of type 2 DN also remains unclear (6–8). Therefore, the aim of the present study was to investigate the role of a sensitive indicator of early renal damage in type 2 DN and identify the cut-off point of type 2 DN among patients with type 2 DM.

## Subjects and methods

### Subjects

A total of 742 Chinese in- and outpatients with type 2 DM (age, 20–75 years) from the project database of Qingdao City People’s Hospital (Qingdao, China) were enrolled in the study. The patients were divided into four groups according to their glomerular filtration rates (GFRs): GFR-A (≥80 ml/min); GFR-B (50–80 ml/min); GFR-C (20–50 ml/min); and GFR-D (<20 ml/min). The patients were also categorized according to their duration of DM, in which ≤1 year duration was considered as a new diagnosis of DM. All the patients satisfied the following inclusion criteria: i) No previous history of liver, kidney or lung diseases; ii) no acute infectious diseases, including urinary tract, respiratory and gastrointestinal infections, during observation; iii) no obstructive nephropathy, glomerulonephritis, renal tubular disease, renal vascular disease, renal tumors, urinary stones, acute and chronic pyelonephritis or other urinary tract infections caused by proteinuria and kidney damage; iv) no medication affecting glucose or insulin metabolism; v) no history of acute cardiovascular and cerebrovascular diseases, including angina pectoris, myocardial infarction and stroke, for the past three to six months; and vi) no various crises and severe diseases during the investigation. The study was conducted in accordance with the Declaration of Helsinki and with approval from the Ethics Committee of Chengyang People’s Hospital (Qingdao, China). Written informed consent was obtained from all the participants.

### Investigation methods

Uniform questionnaires were completed according to the out- and inpatient medical records. The gathered information was reviewed to ensure accuracy, completeness and reliability. Clinical characteristics of the subjects, including age, gender, height, weight, waist circumference, hip circumference, systolic blood pressure and diastolic blood pressure, were also recorded. Uniformly trained researchers performed the investigation.

### Survey content

Survey items included the duration of DM. All the subjects fasted for 8–10 h prior to blood pumping. The levels of various parameters, including SUA, fasting blood glucose (FBG), glycosylated hemoglobin, total cholesterol, triglyceride (TG), high-density lipoprotein and low-density lipoprotein, were determined. FBG was measured using glucose oxidase, cholesterol and other automatic biochemical analyzers. A particle-reinforced projected turbidimetric immunoassay was used to determine the level of serum cystatin C (SCysC), while serum β2-MG (Sβ2-MG) levels were determined using α1-microglobulin (α1-MG) immune scattering nephelometry. Urine specimens were obtained in the morning and used for immune nephelometry to determine the levels of urinary cystatin C (UcysC), urinary β2-MG (Uβ2-MG), UALB and CCr.

### Diagnostic criteria

Diagnostic criteria of kidney damage were based on CRF diagnosis and clinical classification standard were based on the Department of Internal Medicine textbook that was published by People’s Education Press in China (9). The GFR stages were classified as follows: GFR≥80 ml/min, normal kidney function; GFR=50–80 ml/min, initial stage of kidney damage; GFR=20–50 ml/min, decompensated renal function; and GFR<20 ml/min, renal failure. Normal ranges are listed as follows: Blood cystatin, 0.2–1.0 mg/l; blood β2-MG, 1–3 mg/l; blood α1-MG, 10–30 mg/l; UCysC, <0.2 mg/l; Uβ2-MG, 0.1–0.3 mg/l; and UALB, 0–25 mg/l. DM was diagnosed according to the World Health Organization’s DM diagnosis and classification criteria (10).

GFRs were calculated (11) as follows: GFR = 186 × (SCr) − 1.154 × (age) − 0.203 × (0.742 if patients are female), where SCr was expressed in mg/dl, age was expressed in years and weight was expressed in kg. The diagnostic index was calculated as follows: Diagnostic index = 1 − false positive − false negative.

We acknowledge Michael Biotechnology Co, Ltd. for providing Reagent supply during part of the study. The Japanese Wako Pure Chemical Industries, Ltd. (Osaka, Japan) provided reagents which are used to measure SUA, FBG, glycosylated hemoglobin, total cholesterol (TC), TG, high density lipoprotein (HDL), low density lipoprotein (LDL), uric creatine. The instrument was provided by The Japanese Wako Pure Chemical Industries, Ltd. (Osaka, Japan). The Beijing Lidman biochemical Limited by Share Ltd. (Beijing, China) provided reagents used to measure SCysC, UCysC, Uβ2-MG, UALB, β2-MG. The instrument was provided by The Japanese Wako Pure Chemical Industries, Ltd (Osaka, Japan). Bio-Rad (Hercules, CA, USA) provided reagents and instrument which were used to measure HbA1c.

### Statistical analysis

Measured variables are expressed as the mean ± standard deviation. Comparisons between two groups were conducted using the t-test, while multiple group comparisons were performed using univariate variance and covariance analyses. UALB and TG levels exhibited non-normal distributions, thus, these parameters were initially normalized by calculating natural logarithms prior to including these parameters in the multivariate and Spearman’s correlation analyses. The subjects were grouped according to the duration of DM. Multivariate logistic regression analysis was performed to calculate the odds ratios (ORs) and 95% confidence intervals (CIs) of the various stages of renal damage. Receiver operating characteristic (ROC) curve analysis of the various durations of DM was performed to determine the sensitivity and specificity values. The area under the curve (AUC), diagnostic index and maximum duration of the optimal cut-off point were also compared. Statistical analyses were performed using SPSS 19.0 software (SPSS, Inc., Chicago, IL, USA) and P<0.05 was considered to indicate a statistically significant difference.

## Results

### General comparison of the clinical features of the indicators

A total of 742 out- and inpatients with DM (male, 356; female, 386) were included in the study (Table I). An independent samples t-test revealed that the average age of the female group was significantly higher when compared with the male group (P<0.05). In addition, in the female group, the FBG level was significantly lower as compared with the male group (P<0.05). The remaining indicators did not exhibit any statistically significant difference between the gender groups (P>0.05; Table I).

### Comparison of renal function impairment indices

Analysis of variance results revealed statistically significant differences in UCysC, Uβ2-MG and UALB levels when comparing the various endogenous GFR subgroups (P<0.05 or P<0.01) with the control group. However, no statistically significant difference in the SCysC level was identified between the GFR subgroups and the control group (P>0.05). The levels of UCysC, Uβ2-MG, UALB and SCysC in the GFR subgroups were observed in the following order: GFR-C>GFR-B>GFR-A (P<0.05 or P<0.01; Table II).

### Correlation analysis between the various assessments of renal damage indicators

Correlations between renal damage indicators, including GFR, Uβ2-MG, UCysC, UALB, SCysC, SUA, Sβ2-MG and Sα1-MG, were evaluated using multivariate linear analysis. Following adjustment for age and gender, a significant negative correlation was observed between the GFR and level of Uβ2-MG, as well as between UCysC and UALB levels (P<0.05 or P<0.01). The levels of SCysC, SUA and Sβ2-MG exhibited significant positive correlations with the level of CCr (P<0.05 or P<0.01). In addition, UCysC and SCysC levels, as well as Uβ2-MG and Sβ2-MG levels, demonstrated a significant negative correlation (P<0.05). Uβ2-MG and UALB levels also exhibited a significant positive correlation (P<0.05; Table III).

### Cut-off point for DM duration in predicting early renal damage

ROC curves were used to analyze the cut-off points for various durations of DM in kidney damage evaluation. Key indicators included sensitivity, specificity, positive predictive value, negative predictive value, diagnostic index and AUC. The optimum cut-off point for DM was four years, with a diagnostic index of 0.34, sensitivity of 60.79%, specificity of 69.66%, positive predictive value of 55.28%, negative predictive value of 74.23% and ROC AUC of 0.70 (0.69–0.72; Table IV).

### Relative risk analysis between the various durations of DM and kidney damage

To assess the relative risk association, multi-factor multivariate unconditional logistic regression analysis was performed and the OR between the duration of DM and renal damage was calculated. After the values were adjusted for age, gender and smoking history, the subjects were divided into the following groups according to the duration of DM: Newly diagnosed diabetic control group, <1 year (OR, 0.77; 95% CI, 0.67–0.83); group 1, <2 years (OR, 0.79; 95% CI, 0.75–0.85); group 2, <3 years (OR, 0.96; 95% CI, 0.92–1.11); group 3, <4 years (OR, 1.10; 95% CI, 1.02–1.42); group 4, <5 years (OR, 1.74; 95% CI, 1.54–2.00); group 5, <6 years (OR, 1.87; 95% CI, 1.44–2.20); group 6, <7 years (OR, 2.42; 95% CI, 2.03–2.88); group 7, <8 years (OR, 3.51; 95% CI, 2.90–4.24); group 8, <9 years (OR, 4.50; 95% CI, 3.54–5.73); group 9, <10 years (OR, 6.12; 95% CI, 4.44–8.45); and renal damage group, ≥10 years (OR, 9.67; 95% CI, 6.18–15.14). The risk of DN gradually increased as the disease duration increased. Statistically significant differences were observed in the ORs at ≥4 years and as the ORs increased (P<0.05; Table V).

## Discussion

DN is a common complication of DM with an increasing prevalence. In western countries, DN is considered to be one of the leading causes of end-stage renal disease (ESRD) (12). The financial costs of DN and ESRD treatment have increased rapidly, thus, early diagnosis of DN is important to improve the quality of life of patients with long-term DM. The clinical indicators currently used to diagnose DN are SCr, urea and endogenous CCr clearance rates. However, these indicators are easily affected by a number of extra-renal factors, including age, gender, height, muscle mass, diet, body disease conditions and drugs. Therefore, these indicators are not ideal markers of DN. Previous studies have indicated that SCysC can be used as a more efficient endogenous indicator of early DN. However, the sensitivity of SCysC as an alternative indicator of type 2 DN requires evaluation. In addition, the onset time of type 2 DN remains unclear. Thus, the present study aimed to investigate the role of a sensitive indicator of early renal damage in type 2 DN and identify the cut-off point of type 2 DN among patients with type 2 DM (13).

SCysC is a low-molecular weight, non-alkaline, glycosylated protein containing 122 amino acids (14,15). Nucleated cells can produce sustainable CysC that is secreted into the extracellular fluid, including blood, cerebrospinal fluid and semen (16). A previous study indicated that CysC is free from a conventional storage environment and common confounding factors, including gender, age, diet and inflammation; CysC can be determined easily (17). CysC is also an indictor of impaired kidney function. Therefore, this factor is a reliable and sensitive indicator of minor glomerular injuries. CysC concentration rapidly increases at first and then gradually increases as the disease is exacerbated (18). Rigalleau *et al* (19) found that CysC can be used more efficiently than SCr as a marker when early kidney damage is diagnosed among diabetic patients. Furthermore, Willems *et al* (20) showed that CysC is more efficient than SCr as a diagnostic marker for early DN. Additional studies have also indicated that SCysC levels are independent of various risk factors of DM; however, the incidence of type 2 DM may be closely associated with CysC, which is closely associated with DM and DN (21). A clinical study found that the UCysC concentration can be used as an indicator of renal tubular dysfunction (22). The present study found that renal dysfunction aggravated as UCysC, Uβ2-MG, UALB, Sβ2-MG and SCysC levels gradually increased. Diabetic kidney damage exhibits an initial increase in UCysC, Uβ2-MG and SCysC levels, as compared with non-diabetic kidney damage. Each index differently correlates with CCr. The correlation coefficients of UCysC, Uβ2-MG and SCysC were also low. Tubular damage may trigger glomerular damage and tubular and glomerular damages may also co-occur. Our results are consistent with those in additional studies. A previous study revealed that renal disease is not dependent of the onset time or pathogenesis. Researchers also hypothesized the simultaneous or later occurrence of early glomerular lesions in DN with tubular injury. Approximately 12% of patients with DM exhibit high renal tubular protein levels, thereby indicating that tubular damage may occur prior to glomerular lesions (23).

The incidence of microalbuminuria ranges between 14 and 21% in patients with type 1 DM. Microalbuminuria often occurs within the first 15 years of the disease and is considered as a further development and indicator of overt DN (19). However, microalbuminuria is more common in patients with type 2 DM (incidence rate, 26–40%) than in patients with type 1 DM. Although, the onset time in patients with type 1 DM is significantly different. A significant proportion of patients were diagnosed with type 2 DM; this condition was observed prior to the diagnosis of microalbuminuria.

Among newly diagnosed patients with type 2 DM, 14–19% of the patients suffered from microalbuminuria and 2–5% of the patients exhibited overt proteinuria (20). A long-term follow-up (six years) of 278 cases with type 2 DM with or without microalbuminuria showed a significant increase in UALB levels (36.4±1.90–96.9±4.00 mg/l) among patients with microalbuminuria when compared with patients without microalbuminuria (4.20±1.90–8.30±2.80 mg/l) (21). In patients with type 2 DM, the long-term hyperglycemia induced glomerular disease and significantly affected UALB excretion; therefore, DN should be screened. However, the distinction between microalbuminuria and proteinuria in DN diagnosis should be cautiously considered. Microalbuminuria in type 2 DM commonly occurs, thus, can indicate type 2 DM. Microalbuminuria can also be used as a risk factor for cardiovascular disease. The results of the current study indicated that the risk of DN increases with the duration of DM. At ≥4 years, the OR was statistically significant (P<0.05). ROC curves were used to analyze the different durations of DM on the sensitivity, specificity, positive predictive value, negative predictive value, diagnostic index and AUC (Table V) of DN. For DM, a duration of four years had a diagnostic index of 0.34 and exhibited the highest sensitivity (60.79%), specificity (69.66%), positive predictive value (55.28%) and negative predictive value (74.23%). The ROC AUC was 0.70 (0.69–0.72). These results indicated that patients with type 2 DM are likely to suffer from DN within four years, which is earlier than that among patients with type 1 DM (7–10 years). Thus, early intervention and treatment should be administered.

The current cross-sectional study has limitations, thus, the causal association between DM and DN is unable to be fully established. For instance, the variables used in the logistic regression analysis were not continuous. The occurrence or non-occurrence of DN was only used as a dependent variable, which resulted in an incomprehensive analysis. Therefore, a prospective follow-up study is recommended.

In conclusion, UCysC, DM duration and Sβ2-MG can be used as efficient indicators of early renal damage among patients with DM. However, SCysC is not a good indicator of early renal damage. Type 2 DN may initially manifest as tubular damage and gradually progress into glomerular damage. The optimum cut-off point of type 2 diabetic kidney damage is four years. These observations highlight the importance for the early detection and intervention of type 2 diabetic kidney damage. In addition, renal lesions should be determined in glomerular damage and tubular damage.

## Figures and Tables

**Table I:** Clinical characteristics of the patients (mean ± SD).

Clinical features	Male	Female
Cases (n)	356	386
Age (year)	50.18±23.89	53.98±25.60^a^
Weight (kg)	72.51±11.45	62.98±10.09
Duration (year)	5.81±4.89	6.21±5.45
SBP (mmHg)	136.38±18.95	137.22±22.31
DBP (mmHg)	84.73±11.49	81.64±11.58
FPG (mmol/l)	10.72±3.71	9.87±2.61^a^
HbA1c (%)	11.91±4.71	9.97±3.01
TC (mmol/l)	5.45±1.46	5.48±1.44
TG (mmol/l)	0.44±0.61	0.36±0.56
HDL-C (mmol/l)	1.41±0.80	1.49±0.74
LDL-C (mmol/l)	3.19±1.18	3.44±1.18
SUA (umol/l)	335.19±56.18	345.34±54.35

The total number of out- and inpatients with DM was 742, including 356 males and 386 females. An independent samples t-test revealed that the average age in the female group was significantly higher when compared with the male group (P<0.05). In the female group, blood glucose levels were lower than in the male group and the difference was statistically significant (P<0.05). With regard to the remaining indicators, the differences were not statistically significant (P>0.05).

a P<0.05 vs. male group.

SBP, systolic blood pressure; DBP, diastolic blood pressure; FPG, fasting plasma glucose; HbA1c, glycosylated hemoglobin; TC, total cholesterol; TG, triglyceride; HDL-C, high-density lipoprotein cholesterol; LDL-C, low-density lipoprotein cholesterol; SUA, serum uric acid; DM, diabetes mellitus.

**Table II:** Levels of the indicators in the various subgroups (mean ± SD).

Subgroup	Cases (n)	UcysC	Uβ2-MG	UALB	ScysC
GFR-A	264	0.00±0.00	0.22±0.11	18.54±4.24	0.71±0.22
GFR-B	202	3.78±0.52^b^	0.92±0.44^a^	21.35±7.32^a^	0.81±0.35
GFR-C	176	5.23±0.90^b^,^c^	2.67±0.63^b^,^c^	34.04±8.24^a^,^c^	2.81±0.51^a^,^c^
GFR-D	200	8.23±1.74^b^,^d^,^e^	4.29±0.89^b^,^d^,^e^	57.04±11.24^b^,^d^,^e^	4.21±0.64^b^,^d^,^e^

a P<0.05 and

b P<0.01, vs. control group;

c P<0.05 and

d P<0.01, GFR-B, GFR-C and GFR-D groups phase ratio;

e P<0.05, vs. GFR-C and GFR-B groups.

GFR, glomerular filtration rate; Uβ2-MG, urinary β2-microglobulin; Sβ2-MG, serum β2-microglobulin; UCysC, urinary cystatin; SCysC, serum cystatin; UALB, urinary albumin; Sα1-MG, serum α1-microglobulin.

**Table III:** Correlation analysis between the various renal damage indicators.

	GFR	Uβ2-MG	UCysC	UALB	SCysC	SUA	Duration
GFR	1.00	−0.56^a^	−0.86^b^	−0.34^b^	0.49^a^	0.23^a^	0.63^b^
Uβ2-MG	−0.40^a^	1.00	−0.01	0.26^a^	0.03	−0.14	0.21^a^
UCysC	−0.66^b^	−0.01	1.00	−0.02	−0.22^a^	−0.10	0.67^b^
UALB	−0.24^b^	0.26^a^	−0.02	1.00	−0.02	−0.03	0.17^a^
SCysC	0.29^a^	0.03	−0.22^a^	−0.02	1.00	0.00	0.27^a^
SUA	0.23^a^	−0.14	−0.10	−0.03	0.00	1.00	0.20^a^
Duration	0.63^b^	0.21^a^	0.67^b^	0.17^a^	0.27^a^	0.20^a^	1.00

a P<0.05 and

b P<0.01.

GFR glomerular filtration rate; Uβ2-MG, urinary β2-microglobulin; Sβ2-MG, serum β2-microglobulin; UCysC, urinary cystatin; SCysC, serum cystatin; UALB, urinary albumin; SUA, serum uric acid; Sα1-MG, serum α1-microglobulin.

**Table IV:** Cut-off point for DM duration in predicting early renal damage with an ROC curve.

Duration (years)	Sensitivity (%)	Specificity (%)	Positive predictive value (%)	Negative predictive value (%)	Diagnostic index	Area under the curve (95% CI)
≤1	84.45	22.71	37.16	71.98	0.07	0.50 (0.48–0.52)
2	78.19	35.03	38.04	73.86	0.13	0.53 (0.52–0.56)
3	70.64	50.84	39.40	75.29	0.21	0.60 (0.58–0.62)
4	60.79	69.66	55.28	74.23	0.34^a^	0.70 (0.69–0.72)
5	59.88	65.35	40.29	74.13	0.25	0.66 (0.65–0.67)
6	45.41	79.31	40.65	71.88	0.24	0.64 (0.62–0.66)
7	31.32	88.67	40.14	69.43	0.2	0.62 (0.61–0.64)
8	17.76	94.88	38.90	67.00	0.12	0.52 (0.50–0.53)
9	9.13	97.96	37.81	65.48	0.07	0.50 (0.48–0.52)
≥10	3.71	99.38	36.94	64.49	0.03	0.48 (0.47–0.50)

a Optimum diagnostic index.

DM, diabetes mellitus; ROC, receiver operating characteristic.

**Table V:** Relative risk between the various durations of diabetes and kidney damage.

Duration (years)	GFR (OR)	95% CI
<1	0.77	0.67–0.83
1<2	0.79^a^	0.75–0.85
2<3	0.96^a^	0.92–1.11
3<4	1.10^a^	1.02–1.42
4<5	1.74^b^	1.54–2.00
5<6	1.87^b^	1.44–2.20
6<7	2.42^b^	2.03–2.88
7<8	3.51^b^	2.90–4.24
8<9	4.50^b^	3.54–5.73
9<10	6.12^b^	4.44–8.45
≥10	9.67^b^	6.18–15.14

Relative risk adjusted for age, gender and smoking history.

a P<0.05 and

b P>0.05 vs. the 1 group.

GFR glomerular filtration rate; 95% CI, 95% confidence interval; OR, odds ratio.
